# Relationship between exosomes and cancer: formation, diagnosis, and treatment

**DOI:** 10.7150/ijbs.95763

**Published:** 2025-01-01

**Authors:** Chen Huang, Jiajin Li, Zichuan Xie, Xiangjun Hu, Yan Huang

**Affiliations:** 1 Department of Biotherapy, Cancer Center and State Key Laboratory of Biotherapy, West China Hospital, Sichuan University, Chengdu, Sichuan 610041, China.; 2 Sichuan university, West China Hospital of Sichuan University, Chengdu 610041, China.; 3 Health Management Center, General Practice Medical Center, West China Hospital, Sichuan University, Chengdu, China.; 4 State Key Laboratory of Respiratory Health and Multimorbidity, China.; 5 Research Laboratory for Prediction and Evaluation of Chronic Diseases in the Elderly, National Clinical Research Center for Geriatric Diseases, China.; 6 General Practice Research Institute, West China Hospital, Sichuan University, Chengdu, China.

**Keywords:** Exosome, Cancer, Cancer diagnosis, Biomarker, Immunotherapy, Drug delivery

## Abstract

Exosomes are a member of extracellular vesicles. However, their biological characteristics differ from those of other vesicles, and recently, their powerful functions as information molecules, biomarkers, and carriers have been demonstrated. Malignancies are the leading cause of high morbidity and mortality worldwide. The cure rate of malignancies can be improved by improving early screening rates and therapy. Moreover, a close correlation between exosomes and malignancies has been observed. An in-depth study of exosomes can provide new methods for diagnosing and treating tumors. Therefore, this study aimed to review, sort, and summarize such achievements, and present ideas and opinions on the application of exosomes in tumor treatment.

## 1. Background

Exosomes are 40-100 nm extracellular vesicles (EVs) that are actively secreted by almost all cells. They originate from intracellular polycystic bodies and contain proteins, nucleic acids, and lipids. In 1983, Johnstone *et al.* 1,2 studied reticulocyte transformation into erythrocytes and revealed that the budding intracellular body in the erythrocyte plasma membrane further invaginated to form a multivesicular body containing various small vesicles. This multivesicular body fuses with the endoplasmic reticulum or plasma membrane and releases small vesicles outside the cell 3,4. In 1987, Johnstone used the term 'exosomes' to define the substance 5. With a close relationship to the endocytosis system of cells, exosomes are synthesized through three main steps: endocytosis, fusion, and efflux, and are regulated by other factors 6. Exosomes exist and exert essential functions in almost all body fluids, including plasma, serum, saliva, urine, amniotic fluid, ascites, milk, cerebrospinal fluid, nasal lavage fluid, joint cavity fluid, semen, prostatic fluid, bile, and cell culture supernatant 7-9.

Generally, exosomes are essential carriers of information exchange between 'cell-cell' and 'cell-extracellular' environments under physiological conditions 10. Additionally, exosomes regulate several physiological functions through their connotation of biologically active ingredients, including proteins and nucleic acids 11-13. The composition and function of exosomes are closely linked to the cell type from which they originate. Exosomes are involved in tumor development by mediating tumor microenvironment (TME) formation and establishing immune tolerance 14,15. An in-depth study of exosomes can provide new methods for diagnosing and treating tumors. This article initially provides basic information about exosomes, summarizes their association with tumors, and reviews their significance in tumor treatment.

## 2. Exosomes

### 2.1. Structure of exosomes

**Figure 1:**
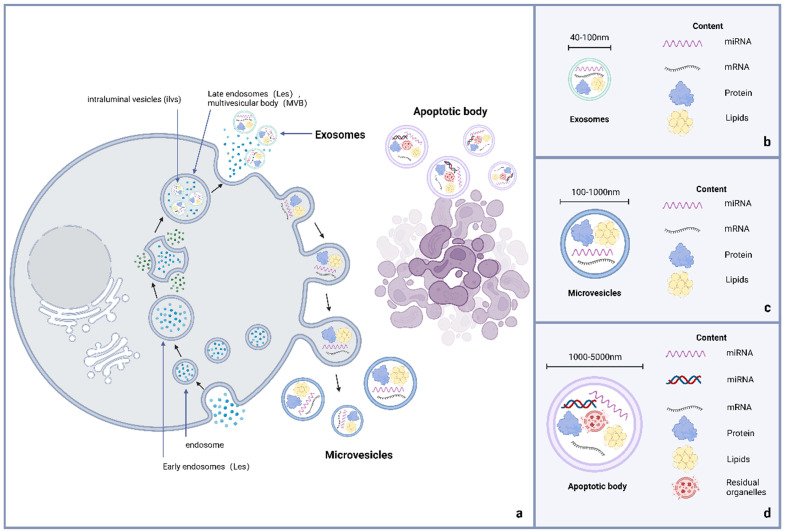
Comparison of exosomes, MV, and apoptotic bodies.

Exosomes belong to the EVs that are subcellular components produced by cell paracrine. The EVs are nanoscale particles that can be divided into exosomes, membrane microparticles, and microvesicles (MV) based on their molecular size and release mode **(Figure 1)** 16,17. Exosomes are EVs with a double-layer membrane structure and diameter of 40-100 nm. Under transmission electron microscopy, they are flat or spherical, often showing a characteristic cup shape. Their density in sucrose solution is 1.13-1.19 g/mL, depending on the cell source and protein content 18-20. The MV, alternatively known as shedding vesicles, are generated by direct budding from the mother cell membrane surface 21. These vesicles typically exhibit diameters of 100-1000 nm, with variations in size observed among individual vesicles. Although MV lack specific surface molecular markers, they can express mother cell-derived surface markers similar to exosomes 22. Apoptotic bodies are microparticles released by programmed cell death or late apoptosis, with large molecular diameters of 1000-5000 nm, distinct from exosomes 23. Moreover, apoptotic bodies contain residual substances of apoptotic cells (including the cytoplasm and organelles) 10,24,25.

Exosomes, MVs, and apoptotic bodies are unique types of EVs. Figure 1a displays the difference in the process of their occurrence. The formation of exosomes is obviously derived from endocytosis, while the occurrence of EVs and apoptotic bodies is linked to cell membrane budding, and apoptotic bodies often contain nuclear components due to the special occurrence links 26. Figure 1b-d distinguish them from the aspects of volume and content.

Exosomes can be considered a double-layer phospholipid molecular layer wrapped in cytoplasmic components. Various membrane protein molecules on the exosome surface participate in their recognition and fusion with recipient cells in addition to exerting their functions. Certain proteins are reliable markers for exosome identification. Exosome capsule components are abundant, including some DNA, messenger RNA (mRNA), microRNA (miRNA), and proteins from mother cells (called source cells). These components are stored in the exosomes, waiting for the 'goods' to be transported to the recipient cells, which exosomes protect to avoid premature degradation by external environmental interference 27-29.

### 2.2. Components of exosomes

The biological components of exosomes are divided into three categories: proteins, nucleic acids, and lipids. As of 2024, the ExoCarta database (http://exocarta.org) contains 286 exosome research results. The identified exosomal components included 9,769 proteins, 3,408 RNAs, 2,838 miRNAs, and 1,116 lipids. Exosomes exhibit heterogeneity owing to their diverse cell sources. This heterogeneity is reflected in various aspects, including size, function, type, and the substances they contain 30. Consequently, exosomes can be divided into two categories based on their biological function. Type I exosomes, which may lack RNA, exhibit immune activity and contribute to antigen presentation and co-stimulation. Type II exosomes are abundant in RNA and promote the exchange of genetic material between cells 31.

#### 2.2.1. Proteins

Exosomes primarily consist of a diverse range of proteins. Exosomal proteins include integral exosomal membranes, lipid-anchored outer membranes, peripheral surfaces, lipid-anchored inner membranes, inner peripheral membranes, exosomal enzymes, and soluble proteins 32. Exosomal proteins can be divided into two categories based on their specificities. One is the ubiquitous proteins in exosomes that participate in their structural composition and are often distributed on the surface or in the lumen. The other is a relatively specific protein linked to the cell sources. The exosome protein content is highly similar to that of the source cells. This means that proteins exist in exosomes, and their contents can vary.

Exosomes commonly contain various proteins, including cytoskeletal componentscomprising tubulin, actin, and microfilament-binding proteins. Additionally, they contain membrane transport- and fusion-related proteins, including Rab 33 and ALG-2 interacting protein X (Alix) proteins 32. Furthermore, exosomes comprise four transmembrane proteins (CD9/63/81/82), which contribute to their production and serve as biomarkers to identify exosomes 33-37. Most exosomes contain heat shock proteins (HSP70/90), antigen-presenting molecules, major histocompatibility complex (MHC I), plasma proteins, cytoplasmic proteins, and nuclear proteins 33,35,38,39. Tetraspanin belongs to the protein superfamily with four transmembrane domains and two extracellular fragments. These compartments are closely associated with various biological functions of the plasma membrane 40,41. Among these exosome proteins, HSP, a molecular chaperone, responds to stress by maintaining proper protein folding and inhibiting protein aggregate formation to avoid histopathological changes 42. Consequently, controlling the biological activity of proteins is a potential method for treating neurodegenerative diseases, Alzheimer's disease, and cancer, among others 43,44.

Proteins related to source cells are relatively specific. For example, exosomes derived from B lymphocytes, dendritic cells, mast cells, and intestinal epithelial cells contain many major histocompatibility complex proteins, MHC I/II 45-48. Exosomes derived from platelets and T cells contain special proteins, including von Willebrand factor, perforin, and granzyme 49. The surfaces of exosomes derived from tumor cells contain tumor-specific protein molecules, including Fas ligand (FasL), tumor necrosis factor-related apoptosis-inducing ligand (TRAIL), transforming growth factor-β (TGF-β) tumor antigens, and immunosuppressive proteins 50,51. This specific protein gives exosomes the potential to be used in the diagnosis, screening, and prognosis of tumors and other diseases. Yoshioka *et al.* 52 proved by comparison that CD63 contained in tumor cell-derived exosomes (TDE) can be used for molecular cancer diagnosis. The Welker team 53 found that an increase in CD81 in the exosomal serum fraction was associated with the activity of hepatitis C and the severity of fibrosis. Nilsson *et al.* 54 isolated exosomes containing biomarkers prostate cancer antigen 3 and transmembrane serine proteases from the urine of patients with prostate cancer, thereby confirming the diagnostic potential of exosomes.

Exosome surface proteins are closely related to the recognition and fusion of exosomes with receptor cells, as well as the specific recognition of exosomes. Four transmembrane proteins exist on the surface of almost all exosomes; therefore, they can serve as biomarkers for exosome identification. Annexins and adhesion proteins are widely present on exosome surfaces and contribute to exosome recognition, docking, and fusion with recipient cells. The peptide-MHC II complex on the exosome surface can stimulate T cells 55,56. Exosome proteins are important carriers of biological information. They are responsible for entering the receptor cells and fulfilling various roles. For example, exosomes derived from antigen-presenting cells can play a role in antigen presentation because they carry MHC II molecules 37.

#### 2.2.2. Nucleic acids

The most common exosomal nucleic acid components are mRNA, miRNAs, and DNA. In 2007, Valadi *et al.* 57 first confirmed the presence of mRNA and miRNA in the exosomes of mouse and human mast cells and discovered that mRNA can be translated into proteins in target cells.

The mRNA is mainly involved in the cell cycle, angiogenesis, and histone modification and is regulated by miRNAs. Exosomal mRNA reflects the state and cytoplasmic content of the originating cells. After entering the cell, mRNA binds to the ribosomes in the cytoplasm and is translated into proteins. Consequently, exosomes affect distal and recipient cell growth through their contents, including participation in stem cell differentiation, organ formation, blood cell production, tumor occurrence, and metastasis. Therefore, mRNA is the main functional exosome component 58,59. However, the RNA delivered through exosomes is not always a natural cellular product. Exosomes released by infected cells contain virus-derived RNA. Upon the uptake of these exosomes by receptor cells, the latter becomes susceptible to infection. mRNA has potential clinical diagnostic significance 60. Lau *et al.* 61 found exosomes in saliva and suggested that they may contain mRNA to diagnose pancreatic cancer.

miRNAs contained within exosomes are transported into the circulatory system alongside them, interacting with recipient cells by binding to them. Subsequently, the miRNA functions as endogenous miRNAs, perform biological processes, and assist exosomes in signal transduction. The modulation of miRNAs can exert effects across different species. For instance, introducing miR-159 mimics consistently enhances breast cancer cell proliferation and reduces myelocytomatosis (MYC) protein levels 62. Body fluids contain secreted miRNAs, which can be detected and extracted. These miRNAs can provide valuable insights into the physiological and pathological states of the body. Consequently, they can serve as markers for disease diagnosis and prognosis 63,64. Characteristic miRNAs secreted by tumor cells can be detected in the serum of patients with cancer. Additionally, detecting virus-secreted miRNAs in patient serum is useful for determining the disease prognosis. Serum exosomal miRNA profiling is an effective non-invasive detection method for the early diagnosis of ovarian cancer 65.

Exosomal DNA (exoDNA) is a cytoplasmic DNA fragment from the nucleus or mitochondria in the case of normal DNA metabolism, accumulation, or both caused by damage and is subsequently excreted from cells with exosomes. It is mainly present in blood circulation and can be integrated into the genomes of specific immune cells. Typically, cells transport damaged DNA into exosomes to maintain their intracellular homeostasis. Additionally, the biological function of exoDNA is more reflected in tumor research. ExoDNA may contain DNA fragments of multiple chromosomes, making it a useful gene repository for tumor cells in the peripheral environment, reflecting the mutation status of the parent tumor cells 66,67. Tumor cell DNA can also use exosomes to complete long-distance metastases.

Tumor cell DNA in exosomes can be used to clinically diagnose tumors. Thakur *et al.* 68-70 demonstrated that stable double-stranded DNA was present in exosomes of cancer cells from various sources, including leukemia, colon cancer, melanoma, breast cancer, lung cancer, prostate cancer (PCa), and pancreatic cancer. This suggests that exosomes and their DNA may serve as potential biomarkers for cancer diagnosis. Circulating-free cell DNA (cfDNA) refers to DNA fragments released into the peripheral blood after physiological processes, including apoptosis, proliferation, or necrosis, and exists as DNA-protein complexes or free DNA. Circulating tumor DNA (ctDNA) is a tumor-derived cfDNA with many cancer-related molecular characteristics, including single-nucleotide mutation and methylation 71-77. Compared with exoDNA, the main methods of ctDNA entering the blood are tumor cell apoptosis and active release, and there is no double-layer membrane structure, resulting in poor stability 78. One of the liquid biopsy technologies is ctDNA detection, which can be employed to monitor gene mutations and effectively diagnose tumors. The results for ctDNA, circulating tumor cells (CTC), and various biomarkers indicate that ctDNA exhibits higher sensitivity and specificity 79-83. Currently, certain challenges impede the clinical application of ctDNA research. These challenges revolve around the need to better understand the biological characteristics of ctDNA and confirm its clinical practicability. Accordingly, ctDNA has not been promoted as a cancer biomarker because of its low concentration in the blood, high fragmentation degree, and weak detection sensitivity compared to the corresponding tissue biopsy 84. Conversely, exoDNA may be a superior biomarker due to its ease of acquisition compared to individual ctDNA 85 and enhanced stability, which are attributed to the larger molecular weight of DNA fragments 86,87. In a liquid biopsy study of advanced tumors, Lazzari *et al.* 88 observed that exoDNA analysis determined the tumor mutation burden more accurately than ctDNA analysis.

#### 2.2.3. Lipids

Common lipids in exosomes include phospholipids, lipid rafts, ceramides, and sphingomyelins. This unique combination of lipid types and contents ensures high exosome stability. Moreover, it facilitates exosome uptake and internalization by recipient cells. Exosome lipids differ from those in parental cells because of their special production pathways. Generally, exosomes contain 2-3 times more sphingomyelin, cholesterol, phosphatidylserine, and sphingolipids than parental cells 89,90. For example, exosomes excreted by human prostate cancer cells-3 (PC-3) contain high sphingolipids, sphingomyelin, cholesterol, and phosphatidylserine levels 91.

Exosomes contain bioactive lipids (including prostaglandins); therefore, exosome lipids participate in many biological processes as signaling molecules. Lipids can participate in exosome formation, maintain their morphology, and affect their functions in the body, rendering them useful for disease treatment.

### 2.3. Formation and molecular regulation mechanism

#### 2.3.1. The formation of exosomes

Exosome formation is closely related to continuous biofilm flow. The cell membrane is invaginated via endocytosis to form endosomes, and multiple endosome fusions can form early endosomes. In the early nuclear endosome stage, the nucleus is acidified, and then the microsomal membrane 'sprouts' inward again to form smaller vesicles, which are stored. The early nuclear endosome develops into late endosomes known as multivesicular bodies (MVB) 92,93. The MVBs are transported to the vicinity of the cell membrane and fuse with it in a calcium-dependent manner, releasing small vesicles with uniform morphology and size outside the cell to form exosomes 3.

In addition to releasing vesicles to form exosomes in the above form, MVBs have two destinations: either they proceed with endocytosis of macromolecular substances or vesicle structures in the cytoplasm followed by fusion with lysosomes to degrade their contents, or they directly fuse with lysosomes and degrade their contents 94,95. Consequently, MVBs can be divided into secretory and degradable MVBs according to their outcomes. The main factor affecting MVB fate is the cholesterol level. Cholesterol-rich MVBs are targeted and sorted to the cell membrane as exosomes, whereas low-cholesterol MVBs are targeted and transported to lysosomes.

#### 2.3.2. Molecular regulation mechanism of exosome formation

Various molecules can regulate exosome secretion, especially the Rab family molecules 96,97. Rab family proteins are the largest small G protein family, and different Rab family proteins are localized on specific cell membranes. Rab protein, a molecular switch for vesicle transport in eukaryotic cells, regulates intracellular vesicle formation and transport. This is achieved by interacting with both upstream regulatory and downstream effector proteins. Consequently, the Rab protein effectively controls the transport and distribution of various proteins within and outside cells. Rab GTPase is a cell membrane receptor that binds to vesicle transport proteins. Specifically, Rab27/35 is liable for MVB transport and docking to the plasma membrane, whereas Rab11 participates in membrane shedding. Ostrowski *et al.* 98 demonstrated that Rab27a mediates MVB docking to the plasma membrane, while Rab27b controls its transfer from the microtubule to cortical actin.

**Figure 2:**
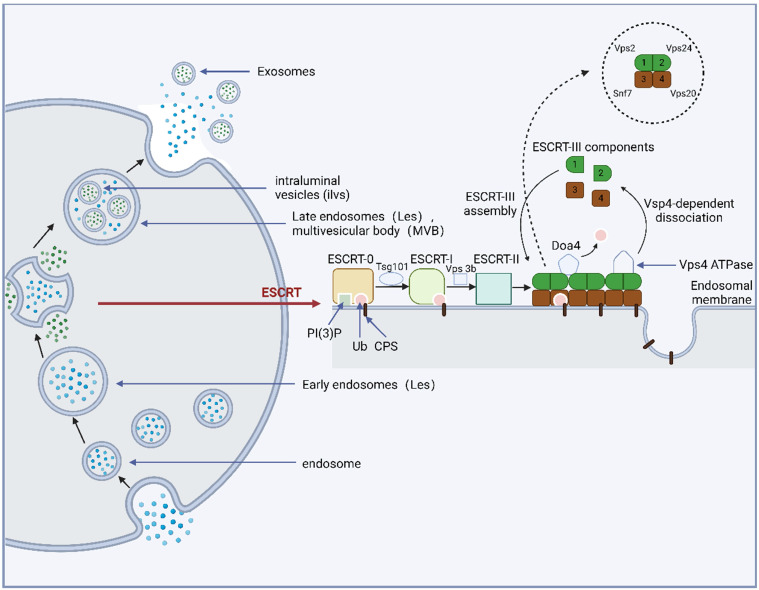
The formation process of exosomes and their main molecular regulatory mechanisms.

MVBs are formed through the endosomal sorting complex required for transport (ESCRT) and an independent ESCRT mechanism 99,100. The main component of the ESCRT mechanism is the ESCRT family, which contains ~20 proteins distributed in four multi-protein complexes (ESCRT-0/I/II/III) and vacuolar protein sorting factor 4 (Vps4), which can recognize and transmit ubiquitinated membrane proteins 55. When cell membrane surface proteins are ubiquitinated, the cell membrane surface and endosome phosphatidylinositol (PI) are phosphorylated to phosphatidylinositol-3-phosphate (PI-3-P) by Vps34 or class III phosphatidylinositol-3-kinase. The membrane region of PI-3-P specifically recognizes and interacts with ESCRT-0. Subsequently, ESCRT-0 binds to the ubiquitinated protein through the zinc finger domain and ubiquitin-interacting motif, enters the MVB, and binds to the tumor susceptibility gene 101 protein (Tsg101) under the action of the Hrs subunit, which can bind to Vps28/36 to recruit ESCRT-II. The cell membrane is invaginated and fixed by the combined action of ESCRT-I/II (which can be described as a supercomplex). Finally, the bud neck was sheared by ESCRT-III to form intracavitary vesicles and MVB. In this mechanism, Snf7/CHMP4 recruits alpha-4 enzyme degradation that removes ubiquitin from MVB inclusions and enables them to enter vesicles 25,101. Before completing deubiquitination, ESCRT-III binds to the Vps4 adenosine triphosphate (ATP) enzyme, leading to its subsequent degradation. Eventually, it recovers to the cytoplasm to complete the recycling of ESCRT-III 102. The ESCRT family of proteins interacts with ubiquitinated proteins and synergistically promotes MVB formation. Moreover, MVB formation involves the entry of selective proteins into these structures. This mechanism provides insights into the protein pathway that enters the MVB **(Figure 2)**.

Many molecules exhibit a regulatory role during exosome formation and allow some substances (RNA and protein) to enter vesicles to complete MVB assembly through ESCRT. The ESCRT family proteins are distributed in ESCRT-0/I/II/III and Vps4, which identify and transport ubiquitinated proteins and promote MVB formation.

Katarina *et al.* 36 identified that certain exosome domains are present in sphingolipid ceramide, and purified exosomes are abundant in ceramide. Other studies also revealed that MVB formation is not entirely dependent on the ESCRT mechanism 103,104. Although the ESCRT mechanism is challenging, many ways exist to promote exosome formation (collectively called the non-dependent ESCRT mechanism). These non-dependent ESCRT mechanisms may be mediated by the accessory protein Alix, which can simultaneously bind to Tsg101 and CHMP4 and participate in exosome formation 105,106. The abundant four transmembrane proteins, CD63-a and ceramide, on the MVB membrane, can induce cell membrane budding and promote MVB formation 36,107-109. Calcium ions (Ca^2+^) accumulate in cells, and intracellular and intercellular pH values, and other factors affect exosome secretion 110,111. By examining exosomes generated by mouse embryonic fibroblasts, Lin *et al.* 112 discovered that Sirtuin6 forms exosomes and mediates protein entry.

The study results on RNA entry into exosomes indicate that there may be similar special sorting sequences in the RNA entering the exosomes, which have a directional effect similar to the cis-acting element and can participate in RNA sorting 113,114. A synaptotagmin-binding cytoplasmic RNA-interacting protein controls mRNA sorting into exosomes 115.

Rab and soluble N-ethylmaleimide-sensitive fusion protein (NSF)-attachment protein receptor (SNARE) protein families promote exosome secretion into the extracellular space. The Rab family, a small GTPase protein group, including Rab11/35, controls the transport of intracellular vesicles, mediates vesicle movement on the cytoskeleton, and targets vesicles to the plasma membrane 116. Overexpression of the Rab GTPase activator protein in mouse oligodendrocytes inhibits Rab by reducing proteolipid protein-loaded exosome secretion. In RNA interference experiments, Ostrowski *et al.* 98 found that Rab27A/B can promote MVB implantation on the plasma membrane and are important molecules for HeLa cells to secrete CD63 exosomes and MHC-II exosomes. SNARE is a protein complex that can promote the fusion of plasma membranes in contact with each other and the fusion of MVB vesicle membranes and plasma membranes in extracellular exosome secretion 117. Downregulating vesicle-associated membrane protein 7, a SNARE family member, in Madin-Daby canine kidney cells inhibits secretory MVB release, preventing the extracellular secretion of exosomes 118,119. Huang presented his research on quantum dot-labeled exosomes (exosome-DNA-QDs complex) and elucidated the potential of the complex for tumor labeling and imaging 120.

### 2.4. The function of the exosomes-important information carrier

This microparticle has been considered a garbage bag discharged by cells to remove metabolic waste 4. Moreover, exosomes are believed to be signaling molecules that suggest cell death or organelle structures of some unique organs 121. Exosomes are also thought to be EV produced by cells to recover surface proteins, thereby regulating signal transduction from the outside to the inside 37. In 1996, a study discovered that exosomes are related to antigen presentation reactions, revealing that exosomes are involved in the biological immune function of the immune system 56. Later, the advancement of protein analysis and other technologies demonstrated that exosomes are specific subcellular structures secreted by living cells. Exosomes possess specific functional activities wherein active substances, including proteins, lipids, mRNA, and miRNA, play a crucial role. However, its primary significance lies in its capacity as an information carrier that facilitates intercellular information exchange.

The primary methods of material exchange between the cells and the external environment are active, passive, and vesicular transport. Recently, vesicular transport has attracted considerable attention. Exosomes regulate material transport and contribute to the transmission of intercellular and intracellular information 10. Exosomes exchange information in three ways. The first method involves the binding of specific surface molecules to target cells. This communication method was first discovered in the field of the immune system. Fas ligand (FasL) interacts with the Fas receptor and initiates T-cell apoptosis by activating the caspase cascade^122^. This information exchangeability depends more on the specific surface signaling molecules of exosomes, which are common in the immune system 123.

**Figure 3:**
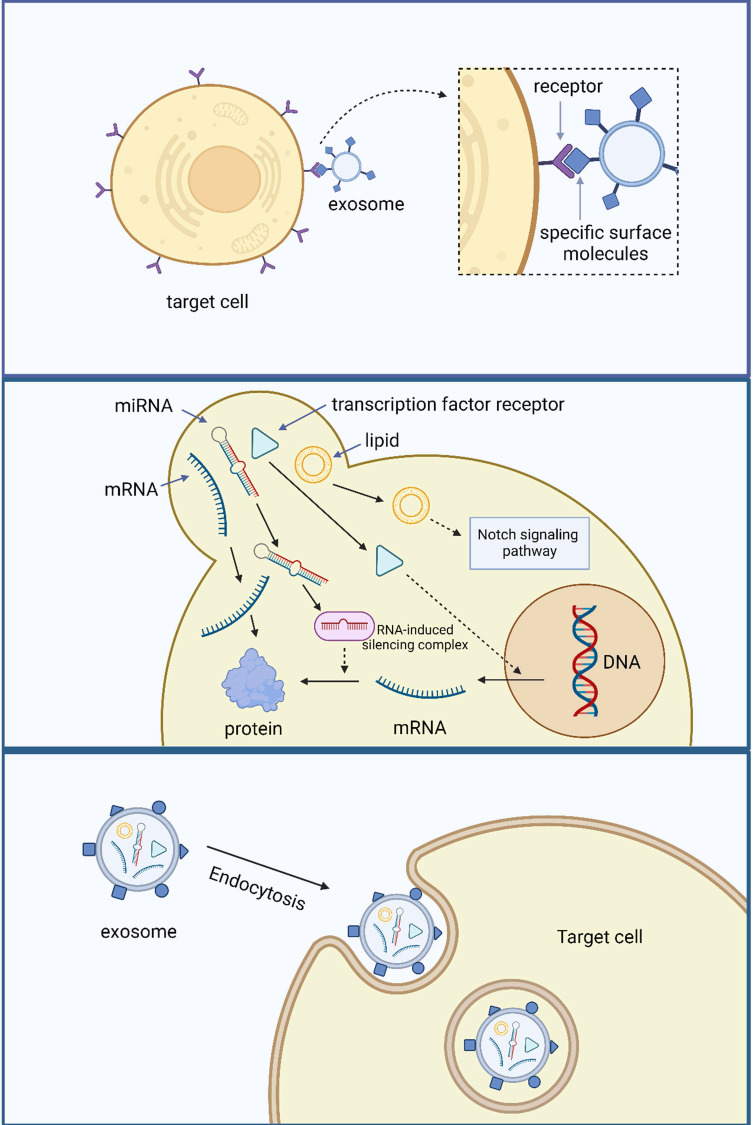
Information exchange function of exosomes.

The second method of exosome exchange information involves direct fusion with the target cell membrane and the release of their contents. In 2007, Valadi *et al.* 57 discovered that human mast cells could capture exosomes produced by mouse mast cells and that their mRNA was translated into proteins after entering the cells. In addition to mRNA, miRNAs can maintain their biological activity and target mRNA translation levels after entering receptor cells 124. This method of information exchange is similar to viral infection and widely exists in different cells throughout various systems of the body, including the tumor cell immunosuppressive mechanism, anti-atherosclerosis mechanism of vascular endothelial cells, fat synthesis of adipocytes, myocardial protection of stem cells, and neuroprotection. In addition to RNA, the biologically active components of exosomes include proteins and lipids. Exosomes isolated from human plasma contain the transcription factor receptor-peroxisome proliferators-activated receptor γ (PPARγ), which regulates the ability of adipocytes and immune-related cells to express genes. Its intercellular transport can regulate receptor cell transcription level 125. The lipid membrane possesses biological properties, and its interaction with tumor cells is observed in the structure of synthetic exosome-like lipid membranes. This interaction can induce apoptosis in human pancreatic tumor cells by modulating the Notch signaling pathway 126. However, Ristorcelli *et al.* 127 observed that the effect of exosomes on apoptosis induction is linked to the degree of differentiation of tumor cells. An inverse relationship exists between the differentiation degree of tumor cells and the expression levels of Notch signaling pathway ligands. Consequently, the impact of exosomes on inducing apoptosis becomes less pronounced as the differentiation degree of tumor cells increases. The third-way exosomes exchange information is that these vesicles are swallowed into the endocytic compartment by receptor-mediated endocytosis or phagocytosis **(Figure 3)** 128.

Exosomes play an essential role in vesicle transport, and the following are its three main mechanisms. In these processes, the stable membrane structure of exosomes enables signal molecules to reach target cells safely.

Exosome-mediated intercellular communication diminishes the significance of the diffusion of information molecules in bodily fluids. This is due to the ability of exosomes to protect and transfer components, including miRNAs and mRNA, which are susceptible to inactivation or degradation in the external environment. Consequently, these components can be securely delivered to the target cells, enabling their participation in regulatory processes. Exosomes transport various bioactive molecules, thereby regulating receptor cells from multiple channels and sites. This process leads to enhanced biological regulation in terms of both degree and precision. Because exosomes are stable and abundant in body fluids, the range and duration of biological regulation mediated by exosomes can also be significantly improved. Accordingly, the characteristics of exosomes as carriers have been applied to treat various diseases (including cancer), which will be discussed later.

### 2.5 Application of exosomes

The main application of exosomes is their strong association with the origin cells, rendering them an efficient biomarker. Almost all cells secrete exosomes into body fluids, making exosomes accessible biomarkers. Additionally, miRNAs are easily degraded by enzymes or other environmental factors in bodily fluids. However, exosomes can protect carried miRNAs, enabling miRNA extraction and detection as biomarkers. Tumor cells secrete more exosomes than normal cells, rendering them as diagnostic biomarkers for cancer. TDE isolation and identification in body fluids detects and monitors cancer and precisely identifies DNA, RNA, proteins, and other biomolecules without contamination by non-TDE 68. Melo *et al.* 129 studied miRNAs in exosomes derived from breast cancer cells.

Consequently, these miRNAs have been proposed as biomarkers for screening breast, lung, and other cancers 130,131. In a study on Alzheimer's disease, phosphorylated tau protein was found to be transmitted through exosomes. Exosomes express amyloid b protein in characteristic plaques; accordingly, the characteristic proteins of exosomes are also involved in plaques 132,133. Ebrahimkhani *et al.* 134 conducted a study wherein they isolated exosomes from serum samples and observed that specific miRNAs within these exosomes could potentially influence the development and progression of multiple sclerosis, including its clinical manifestations and disease course. Exosomes are also closely linked to diabetes type I and II 135-138. Although exosomes can be used as biomarkers to diagnose diseases, limitations still exist, including difficulty in sample acquisition and preservation and low specificity of biomarkers 137.

## 3. Exosomes and tumors occurrence and diagnosis

### 3.1. Changes of exosomes in tumor

Tumor detection methods include pathological diagnosis, imaging, endoscopy, and tumor marker (TM) examination. Although they can complement each other and give full play to their respective strengths, they still have shortcomings. The presence of tumor heterogeneity poses a challenge in accurately assessing the overall tumor state based on biopsy results from a single specimen. Pathologists tend to prioritize the examination of advanced or more severe tumor tissues during sampling, which can lead to the oversight of progressing and invasive tissues, thereby compromising the detection process. Similarly, immune markers are heterogeneously expressed in tumors; consequently, immunohistochemical results are incomprehensive. Imaging examinations involve many techniques, including computed tomography, magnetic resonance imaging, and ultrasonography. However, detecting malignant cells becomes challenging when the quantity is limited to a few cells. The test results can only be obtained when a sufficiently large number of tumor cells are present in the tissue to create observable changes in imaging. Physicians must carefully consider the successive application of endoscopic examinations due to their invasive nature despite the various diagnostic indications they offer. Multiple TMs have been identified and can be used to detect tumors, observe curative effects, and evaluate prognoses. However, TM examinations can only assist in tumor diagnosis because most TMs exist in malignancies, benign diseases, embryonic tissues, and even normal tissues. Consequently, some TM contents may be abnormal and normal when benign diseases occur, even in the presence of malignant tumors.

Accordingly, exosomes and related molecules can change according to the original cell state; the tumor cell-secreted exosome levels are higher with altered exosome components 9,37,129,139. Additionally, exosomes play a protective role in their inclusions by preventing miRNAs and other substances from being degraded by extracellular environmental factors, thus losing their biological activity. As a result, exosome detection can be an innovative, non-invasive tumor diagnosis, and more TM with higher sensitivity and specificity can be proposed to improve related research.

#### 3.1.1. Breast cancer

The increasing incidence of breast cancer renders it the most common cancer. Based on histopathology, breast cancer can be divided into triple-negative breast cancer (TNBC) and non-TNBC types. The most invasive subtype, TNBC, has poor prognosis, enhanced metastasis, and a high recurrence rate. TNBC lacks estrogen receptors, progesterone receptors, and human epidermal growth factor receptor 2 (HER2).

Through TME regulation, breast cancer-derived exosomes can alter the expression of genes associated with cell proliferation, apoptosis, and invasion. This, in turn, develops drug resistance and promotes tumor growth. For example, exosome-mediated miR-10b secretion inhibits the protein levels of its target genes (HOXD10 and KLF4) and enhances breast cancer cell invasion 140. Moreover, tumor-secreted exosomal miR-9, known as a metastasis-promoting miRNA, is upregulated in various breast cancer cell lines. This miRNA can be internalized by normal fibroblasts and transformed into a cancer-associated fibroblast phenotype, promoting tumor growth 141,142. miR-105 can downregulate ZO-1 in endothelial cells and promote lung and brain metastases in breast cancer 15,143. The levels of carcinoembryonic antigen (CEA) and cancer antigen (CA) 153 in circulating exosomes are associated with cancer progression 144,145. Compared to exosomes from healthy individuals, exosomes from patients with breast cancer have higher miR-21 and miR-1246 expression 146. Exosomal miR-101 downregulation correlates with lymph node-positive cancers. Meanwhile, miR-373 levels were significantly upregulated in triple-negative, estrogen receptor (ER)-negative, and progesterone receptor (PR)-negative breast cancer patients **(Table 1)** 147.

#### 3.1.2. Lung cancer

In terms of tissue classification, lung cancer can be divided into two types: non-small cell lung cancer (NSCLC) and small cell lung cancer, with the former having a higher incidence 149. Currently, early lung cancer treatment involves radical resection with or without radiotherapy and chemotherapy, which has shown positive results. However, the early detection rate of lung cancer is low because the symptoms are unclear. Accordingly, lung cancer is diagnosed at an advanced stage, increasing the treatment difficulty and recurrence risk 150. Despite the use of molecular-targeted therapies, the lung cancer mortality rate has significantly increased. A fundamental problem is the failure to improve the early diagnosis rate. Consequently, exosomes can serve as biomarkers for early lung cancer diagnosis and good prognosis **(Table 2)** 151. Cazzoli *et al.* 152 demonstrated that miR-378 and miR-200b-5p in circulating exosomes have high sensitivity and specificity for lung cancer diagnosis. Several exosome biomarkers, including miR-146a-5p and miR-486-5p, are preferred for early NSCLC diagnosis 153. The prognostic effect of lung cancer can be detected using epidermal growth factor receptor (EGFR) 154. Adenocarcinoma and squamous cell carcinoma can be distinguished based on miR-181-5p, miR-30a-3p, miR-30e-3p, miR-361-5p, miR-15b-5p, miR-10b-5p, and miR-320b 155. The miR-320b levels can be used to evaluate the PD1 therapeutic effect on NSCLC 156.

#### 3.1.3. Colorectal cancer (CRC)

The CRC is the most common malignant tumor of the digestive system. Radical resection is the most effective treatment, whereas other methods often have little effect. Currently, the blood biomarkers used are CEA and carbohydrate antigen 19-9. However, its sensitivity and specificity are low, and its diagnostic efficacy is suboptimal.

Biomarkers that can be used for early CRC diagnosis, including miR-125a-3p, miR-320c, and miR-17-92a, have been identified and proven in CRC-derived exosomes 168,169. Circulating long-chain non-coding RNA (lncRNA) can be used as a molecular marker because it is correlated with CRC development, invasion, and metastasis **(Table 3)** 170. The lncRNA urothelial carcinoma-associated 1 is an RNA regulator of CRC progression as it controls the competing endogenous RNA network. The increased expression of this factor indicates the active proliferation of tumor cells and suggests their increased resistance to cetuximab 171.

#### 3.1.4. PCa

PCa ranks second among all-male malignancies and first among male genitourinary system malignancies. The therapeutic effect of cancer depends on the degree of development; therefore, accurate early diagnosis is crucial. Prostate-specific antigen is a commonly used PCa screening marker but cannot accurately distinguish between benign and malignant tumors.

The biological molecules mediating intercellular communication in PCa cell-derived exosomes are mainly RNA, and the has-miR-101-3p expression level can be used to identify PCa and benign diseases. Circulating exosomal messenger RNA (exosomal mRNA, emRNA) differs significantly from mRNA in tissue cells and can be used to diagnose PCa. miR-1290 and miR-375 can be used as prognostic biomarkers for castration-resistant PCa 190; miR-423-3p can be employed as a biomarker for predicting castration-resistant PCa **(Table 4)**. The established PCa metastasis risk score model can predict and evaluate PCa prognosis by combining urinary exosome indicators 191. The levels of integrin αvβ3 and CD9 in plasma exosomes of PCa patients are higher than those of healthy individuals, which can be used to track PCa progression 192.

### 3.1.5. Gastric carcinoma (GC)

The most common malignant tumor in humans worldwide is GC, the incidence of which is declining, yet remains prevalent. The prognosis of patients with GC is poor, and the survival rate is lower in patients with advanced stages or recurrence. Shen *et al.* 195 reported that deleting lysine-specific demethylase 1 can reduce PD-L1 and restore the T-cell response of GC, which provides a novel concept for GC immunotherapy. Zhang *et al.* 196 blocked GC cell proliferation, migration, and other activities by circNRIP1 knockdown, proving that circNRIP1 can transmit information between GC cells through exosomes and promote tumor metastasis *in vivo*.

#### 3.1.6. Other cancers

Other cancers are associated with exosomes during their progression, metastasis, and invasion. Exosomes secreted by pancreatic cancer cells regulate tumor extracellular matrix (ECM) composition, cell-cell information exchange, and cell migration, in addition to promoting pancreatic cancer progression. The miRNA in exosomes can be a potential biomarker for pancreatic cancer due to the significant correlation between its metastasis and late-stage and high miR-17-5p levels 197. Glypican-1 (GPC1)-positive circulating exosomes can diagnose early and advanced pancreatic cancers with extremely high accuracy and sensitivity 68. Vesicle bodies isolated from 143 B human osteosarcoma cells contain various substances that affect the TME, involving matrix metalloproteinases (MMPs). MMP-1/13 is important in ECM remodeling 196. Exosomes are also crucial for diagnosing nervous system tumors, and cerebrospinal fluid is a reliable source of biomarkers 198. Liao *et al.* 199 found that exosomal miR-21 was associated with the recurrence and distant metastasis of esophageal cancer. Four transmembrane proteins, CD-9, were detected in the TME of 143 B human osteosarcoma. It is a specific marker of exosomes and a membrane fusion protein of precursor osteoclasts that regulates the differentiation and maturation of osteoclasts. Its overexpression promotes the homing of cancer cells and induces bone resorption in osteoclasts. Inhibiting exosome production can effectively regulate the TME of osteosarcoma and block bone destruction **(Table 5)** 200-202.

### 3.2. The role of the TDE

**Figure 4:**
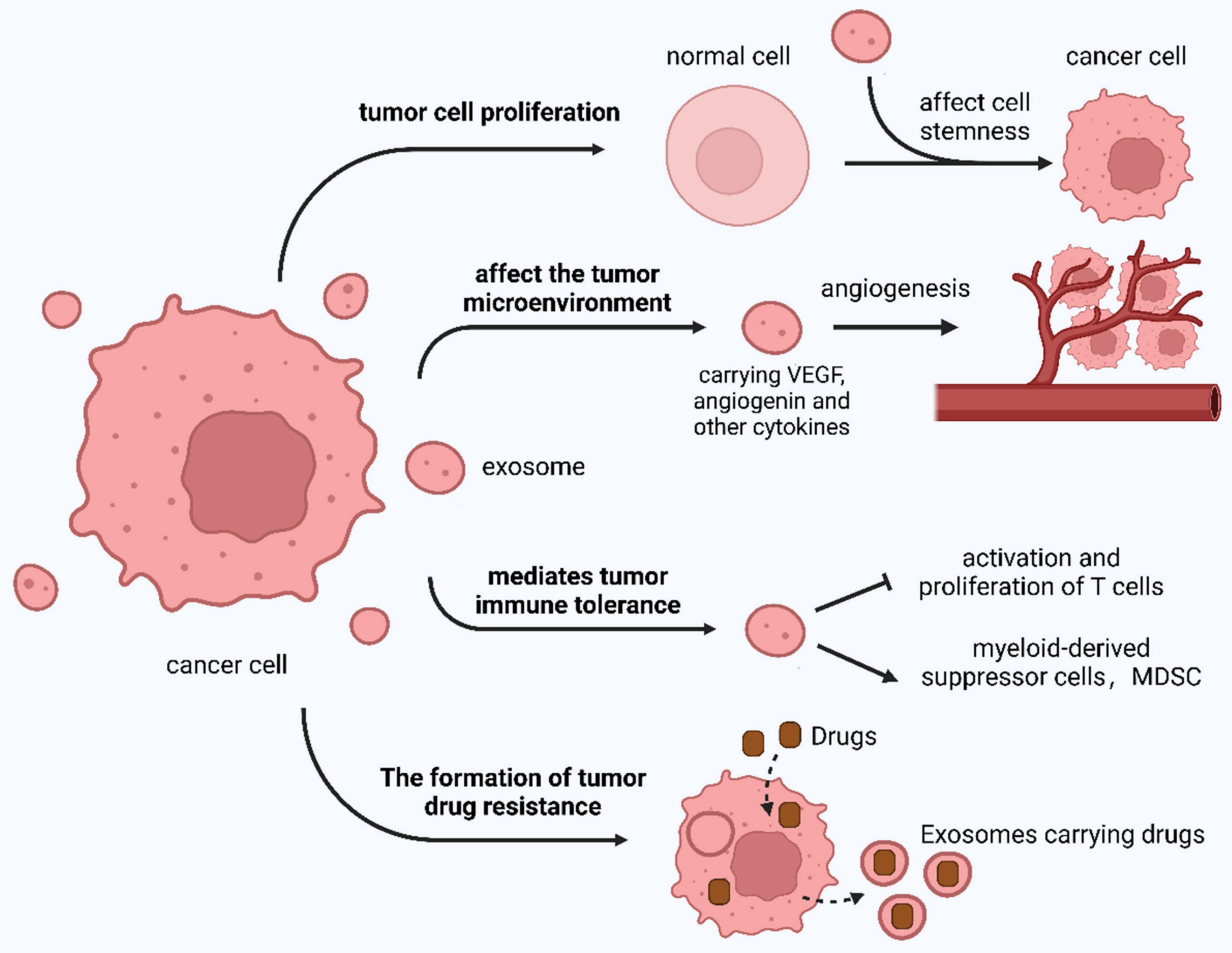
The role of the TDE.

The TDE regulates tumor occurrence and development in addition to regulating the effects of tumor treatment in various ways **(Figure 4)**.

The TME is significantly influenced by the TDE, which affects various aspects of tumor biology. These include tumor progression and invasion, TME alterations, tumor immunity modulation, and resistance development to anticancer drugs.

#### 3.2.1 TDE mediates the proliferation and stemness formation of tumor cells

Exosomes affect tumor cell stemness and promote their development by upregulating Notch, Wnt, and other signaling pathways 129,204,222. Exosomes secreted by highly metastatic melanoma cells can induce bone marrow progenitor cells through MET receptors and increase the metastasis of primary tumors 223. Exosomes derived from breast cancer cells contain molecules comprising argonaute protein 2 (AGO2) and TAR RNA-binding protein (TRBP), which can promote the production of mature miRNAs and induce tumor formation 129.

#### 3.2.2 TDE affects the TME

 The TME is a local pathological environment comprising tumor cells, endothelial cells, cancer-associated fibroblasts, inflammatory cells, and ECM, which are conducive to tumorigenesis, development, and metastasis 224. Exosomes are particularly abundant in the TME and are closely correlated with tumor occurrence and development, immune escape, and microenvironment.

With the deepening of tumor immunology research, multiple immunosuppressive cells were found to comprise regulatory T cells, myeloid-derived suppressor cells, and tumor-associated macrophages in the TME 225. These cells and their secretion-related factors inhibit the body's anti-tumor immune response, constituting an immunosuppressive local environment. This environment is conducive to tumor cells escaping the immune response and is a major obstacle to effective tumor treatment. Exosomes regulate the biological functions of receptor cells, which are important carriers of intercellular information transmission. Research indicates that when tumors occur, normal and tumor cells mediate the survival of these immunosuppressive cells, secretion of effector substances, and accumulation of local tumors through the secretion of exosomes, which is conducive to immunosuppressive TME formation.

TDE uptake by endothelial cells induces angiogenesis. Moreover, exosome synthesis increases under hypoxic conditions, leading to heightened angiogenic activity 226-228. This process has been demonstrated in breast cancer cells, where hypoxia can trigger exosome secretion. TME acidification is also conducive to exosome transport, significantly stimulating angiogenesis, similar to breast cancer cell exosomes 229.

TDE can be absorbed by stromal cells and alters the microenvironment to facilitate tumor growth 204. Exosomes contain or activate cytokines, including angiogenin, fibroblast growth factor (FGF), and vascular endothelial growth factor (VEGF), to promote tumor blood vessel formation, with FGF and VEGF exerting synergistic effects 230,231. Hypoxia-derived exosome circR-133 is transported to normal cancer cells and promotes cell migration through the miR-133a/GEF-H1/RhoA axis. TDE can carry MMPs, A disintegrin, and metalloproteinases 232,233 to degrade the ECM, cut intercellular adhesion molecules, and promote tumor cell metastasis 234-238. In 2015, Costa-Silva *et al.* 204 discovered that by modifying pre-metastases, pancreatic cancer cells can increase metastasis possibility.

PCa cell-derived exosomes can affect the TME by affecting fibroblast activation, promoting tumor angiogenesis, and participating in tumor immunity. These exosomes can stimulate tumor-associated fibroblasts to acquire carcinogenic properties and increase drug resistance to CRC 129,239.

Additionally, tumor cells can manipulate the host immune function, and the body can transport tumor-specific miRNAs to immune cells through exosomes so that tumors can escape immune escape, providing a new idea for tumor immune checkpoint therapy.

#### 3.2.3 TDE mediates immune tolerance of tumor cells

TDE is an antigen-presenting system and a source of tumor rejection antigens 144,240. Immune tolerance induced by TDE is associated with surface FasL. Fas is present on various cell surfaces. When external stimuli stimulate the body, Fas is rapidly upregulated and induces cell death through the Fas/FasL pathway, thereby regulating the immune response and maintaining T cell tolerance 241. Specific types of TDE can express PD-L1 and directly inhibit the proliferation and activity of T cells by binding to PD-1 in T cells 242. Chen *et al.* 243 found that exosomes carrying immunogenic molecules released by mouse B-cell lymphoma under heat shock conditions could induce DC maturation, thereby activating the response of CD4^+^/8^+^ T cells to tumors.

#### 3.2.4 TDE mediates chemotherapy resistance of tumor cells

TDE mediates epithelial-mesenchymal transition (EMT) and intracellular drug rejection in tumor cells 244,245. The proteins transported by exosomes enhance fibroblast growth, hinder anti-tumor drug transport, and upregulate tumor drug resistance 246. This drug resistance also increases because of exosomal mRNA and miRNA 247. Safaei *et al.* 248 have demonstrated that exosomes released by cisplatin-resistant ovarian cancer cells carry numerous cisplatin. Consequently, tumor cells can upregulate exosome release to expel more anti-tumor drugs. Shedden *et al.* 249 captured various anti-tumor drugs that were encapsulated and excreted by exosomes using fluorescence microscopy. This has been observed in various tumors, indicating that exosomes affect drug resistance in tumors.

## 4. Exosomes and tumor therapy

### 4.1. Tumor therapy associated with small molecule drugs

Traditional cytotoxic drugs (chemotherapy drugs) and molecular-targeted drugs are small-molecule drugs. Chemotherapeutic drugs are rarely used alone in clinical practice. However, depending on the actual situation, they are combined with surgery, radiotherapy, or biotherapy to maximize the cure rate of patients. Targeted drugs have high selectivity and low cytotoxicity, and their mechanisms of action are novel and effective. A problem closely linked to small-molecule drugs in clinical practice is the development of drug resistance. With the deepening of research on exosomes, the mechanism of drug resistance in some tumors has gradually become clear.

Drug-resistant breast cancer cells may spread drug resistance and change chemosensitivity in receptor-sensitive cells through intercellular metastasis of exosomal miRNAs (miR-100/222/30a) 250,251. The intercellular transport of P-glycoprotein (P-gp) develops drug resistance through drug transmembrane efflux. Exosomes mediate drug resistance by delivering P-gp from docetaxel-resistant cells to sensitive cells 252-254.

In ER-positive breast cancer, exosomes secreted by tamoxifen-resistant cells (MCF-7TamR) can significantly promote proliferation and colony formation of tamoxifen-sensitive cells (MCF-7wt) in the presence of tamoxifen. This is partially attributed to exosome-encapsulated miRNAs (miR-221/222). Subsequently, miR-221/222 downregulated the protein levels of its target genes, P27 and ERα, thereby increasing recipient cell resistance to tamoxifen^255^. HER2-positive exosomes from BT-474 and SK-BR-3 cells interfere with the biological activity of HER2-targeted therapeutic drugs by directly binding to trastuzumab and preventing its anti-proliferative effects on cancer cells. In contrast, the negative effect on the anti-proliferative activity of lapatinib was almost non-existent 256-258. Paclitaxel-loaded exosomes showed strong anti-proliferative activity against the human pancreatic cell line CFPAC-1 259.

Curcumin can inhibit miR-21 transcription and exhibit anticancer properties, but its application is limited due to its water solubility and poor stability 260. Doxorubicin is a highly effective anticancer chemotherapeutic drug; however, its excessive cardiotoxicity limits its clinical application 261. Applying exosomes to load and transport these drugs can compensate for their shortcomings, significantly improve bioavailability, and enhance anti-tumor effects 262,263.

### 4.2. Tumor treatment related to tumor immunity

The exosome surface carries MHC I/II and CD9/63 proteins. When exosomes are the tumor source, their proteins contain tumor-specific antigens. Dendritic cells can recognize and process antigens carried by TDE and activate T lymphocytes. TDE also stimulates natural killer cells 264. In addition to its immune activity, TDE plays an immunosuppressive role. Immunotherapy plays a significant role in tumor treatment.

#### 4.2.1. Tumor vaccines

The basic principle of tumor vaccines is to use tumor antigens to induce the body to produce specific anti-tumor immune responses through active immunity, stimulate the immune protection mechanism, and achieve the purpose of treating tumors or preventing their recurrence. Proteins carried by naturally occurring exosomes, including Tsg101, Alix, Rab, CD63/81, and Hsp90, can transmit information and regulate immune responses, indicating that exosomes can help develop new cancer vaccines 265,266. Sedlik *et al.* 267 transfected the pcDNA3.1 plasmid carrying the ovalbumin (OVA) antigen gene and the lipid binding domain gene of milk fat globule epidermal growth factor (EGF) factor VIII (MFGE8) into HEK-293T cells. Subsequently, the cells were cultured to prepare exosomes and immunized mice. The results disclosed that these exosomes could induce OVA-specific CD^4+^/^8+^ T-cell responses. OVA-pulsed immature dendritic cells (iDC)-derived exosomes can also directly activate OVA-specific CD^8+^ T-cells. Moreover, after mature DCs take up the MHC-antigen complex carried by iDC-derived exosomes, they can present antigen processing to T cells to promote T cell activation. DC-derived exosomes can directly present antigens, activate immune cells, and serve as carriers for delivering MHC-antigen complexes to induce immune responses 268-270.

#### 4.2.2. Immunomodulator

Immunomodulators are non-specific biological products that regulate immune function. They can be classified into immune enhancers, immunosuppressants, and bidirectional immunomodulators. Palmitoylated proteins on the surface of breast cancer cell-derived exosomes can stimulate the activation of NF-κB in macrophages, releasing pro-inflammatory factors, including interleukin-6 (IL-6), tumor necrosis factor-α (TNF-α), and chemokine (C-C motif) ligand 2, and enhance the body's immune function 271. Some exosomes strongly inhibit the immune system. When TDE is co-cultured with T cells, it inhibits CD3 and Janus kinase-3 expression, inducing apoptosis 272. Given the significant involvement of exosomes in cancer-related mechanisms, inhibiting exosome production and release, as well as reducing exosome absorption by recipient cells, has the potential to exert an anti-tumor effect 273. Syndecan-syntenin-ALIX is a vital signaling pathway that regulates exosome biosynthesis and may be a therapeutic target for reducing exosome release 274. An investigation on the immune response of TD exosomes revealed that exosomes trigger NF-kB activation in macrophages, resulting in increased secretion of pro-inflammatory cytokines (IL-6, TNF-α, and GCSF) 275.

#### 4.2.3. Adoptive immunotherapy

Adoptive cellular immunotherapy, also called somatic cell immunotherapy, involves the extraction of normal cells from the peripheral blood of patients. These cells are then cultured *in vitro* to induce their activation and proliferation. Subsequently, cultured cells are administered to patients to induce tumor cell death, regulate the body's immune function, or both. Ultimately, the objective of this therapeutic approach is to treat tumors.

Chimeric antigen receptor T (CART) cell immunotherapy is a promising cancer treatment option. T cells can recombine with specific antigen receptors using viral vectors. As a consequence, T lymphocytes target tumor-associated antigens more precisely, leading to more effective cytotoxic responses. However, CART cells can expand uncontrollably. Besides, two-thirds of patients may develop cytokine release syndrome (CRS) ten days after infusion of CART cells due to uncontrolled cytokine release by modified T cells 276. Exosomes produced by CART cells can avoid CRS because these exosomes can retain the therapeutic ability of their source cells and control the continuous expansion of CART cells in the body. This therapy is effective only for blood diseases (including lymphoma). Owing to their small exosome size, CART cell-derived exosomes can easily cross the blood-brain barrier or tumor cell membrane, making it feasible to apply this therapy in other tumor treatments 277.

### 4.3. Tumor therapy related to drug delivery

Traditional chemotherapeutic drugs often have drawbacks, including poor solubility, easy clearance by the human body, poor biocompatibility, less distribution of target tissues, and low cell permeability. Many attempts have been made to identify carriers that transport drugs to target cells to ensure their effectiveness 278. As natural liposomes, exosomes have the following characteristics that suggest that they are high-quality drug carriers: they (1) are widely present in human body fluids, showing good stability in blood circulation 279, (2) are rich in inclusions, proving that they can carry most biomolecules, (3) have directional homing ability and can enhance their cell-specific targeting effect by artificially modifying biofilms 280, and (4) can pass through the blood-brain barrier 281. Moreover, drug carriers for tumor treatment often require low immunogenicity and toxicity, and the adverse immune response caused by exosomes is extremely weak 282,283. Using macrophage exosomes to load paclitaxel (PTX) in treating lung cancer has shown efficacy 284.

Commonly selected drugs include small-molecule drugs (PTX and platinum) or biomolecular drugs (proteins and genes). The methods of introducing drugs into exosomes are as follows: (1) chemical transfection or co-culture of the mother cells; exosomes secreted by the mother cells can directly load drugs. However, this method is limited because it requires that the drugs do not cause excessive damage to mother cells 285,286. (2) Exosomes are chemically transfected or co-cultured with the contained drugs. Since there is no suitable method to separate exosomes from the transfection agent, it is impossible to explain whether the effect is from the transfection agent or exosomes, whether it is a drug in exosomes, or a drug attached to the surface of exosomes 287,288. (3) By applying an electric field to the exosomes, the phospholipid bilayer is broken down to produce a cavity that can be repaired, and drug molecules are introduced into the exosomes under the action of an electric field force to achieve drug loading. However, this may affect drug integrity 289,290.

The routes of administration are also abundant, and the common ways are as follows: (1) Intravenous injection: this is the most extensive route of administration. Intravenously injected exosomes are delivered to the brain and pancreas, especially to tumor tissues 291,292. (2) Intratumoral injection: ensuring specific delivery of drugs, avoiding damage caused by invasive surgery, and successfully reducing tumor volume 293,294. (3) Nasal administration: entering the brain parenchyma through the blood-brain barrier non-invasively through the nasal cavity and inhibiting brain inflammation and cancer 293,295,296. (4) Orally: exosome-like vesicles extracted from grapes can induce intestinal stem cell proliferation and promote enteritis resolution 297. (5) Intraperitoneal injection: curcumin-containing exosomes can increase the anti-infective activity of curcumin after intraperitoneal injection 298.

This treatment also received good feedback. Pascucci *et al.* 259 co-incubated high-dose PTX with mesenchymal stem cells (MSCs) to induce MSC-derived exosomes for PTX loading. This effectively inhibited the proliferation of pancreatic cancer cells. Kim *et al.* 299 used the Lewis lung cancer mouse model and confirmed that the exosome-PTX complex showed a stronger inhibitory effect on tumor metastasis and progression than free PTX. Shamili *et al.* 300 used non-viral vectors to introduce plasmids encoding TRAIL-GFP into MSCs. The results showed that MSCs-derived TRAIL-loaded exosomes inhibited melanoma progression by promoting necrosis of cancer cells, and its anti-tumor activity was dose-dependent.

In addition to drugs, exosomes can act as carriers of specific nucleic acids in gene therapy 57. Exosome-small interfering RNA (siRNA) complexes are gradually being used in tumor therapy. A small double-stranded non-coding RNA, siRNA, comprises 20-30 nucleotides and can target complementary mRNA through RNA interference mechanisms, leading to mRNA degradation and corresponding gene silencing. However, siRNA is susceptible to other enzymes, and its combination with exosomes can improve its stability and enhance efficacy 301. Head and neck cancer (HNC) usually has poor prognosis. One reason is that EMT makes cancer cells more invasive and metastatic, and transient receptor potential polucustic2 (TRPP2) is an ion channel that can regulate EMT 302,303. Wang *et al.* 303 studied whether TRPP2 siRNA could knock down TEPP2 and affect HNC growth and metastasis. They prepared a TRPP2 siRNA-exosome complex and designed experiments to demonstrate that free TRPP2 siRNA and the TRPP2 siRNA-exosome complex can downregulate TRPP2 in cancer cells, inhibit EMT, and significantly weaken tumor cell invasion and growth.

The treatment plan is dynamic. Consequently, thorough consideration is necessary in choosing cell-derived exosomes for various pharmaceuticals, considering the individual conditions, and placing significant importance on customizing the suggested approach. For example, exosomes secreted by tumor cells can better home to the tumor site when used as drug carriers. However, there is a risk of promoting tumor growth and immunosuppression 304. Exosomes secreted by macrophages have a good inflammatory tendency and can cross the blood-brain barrier for treating brain diseases. Exosomes secreted by dendritic cells have good immune effects 305. Accordingly, selecting exosome source cells is the premise for achieving the best therapeutic effect.

## 5. Conclusions

Oncology is crucial in clinical medicine, and researchers have endeavored to understand the progression mechanism through various approaches to explore more efficient and safer diagnosis and treatment methods. Exosomes promote tumor progression, metastasis, and invasion through their effects on TME. Additionally, exosomes develop drug resistance in tumors by expelling drugs from the tumor cells. Moreover, exosomes can bidirectionally modulate the immune activity of tumors. Therefore, we attempted to understand the tumor from the exosome perspective and summarize the existing research results to support and help clinical workers.

Exosomes are mainly used in TM examination in tumor diagnostic species, and some existing biomarkers that can be used for diagnosis are summarized in a table. It is noteworthy to mine more tumor biomarkers and extract exosomes more efficiently for tumor diagnosis.

Currently, treatment regimens for drug delivery have reached maturity; however, shortcomings remain. Although exosomes have natural targeting ability, their strength is low. Consequently, in some cases, they must be artificially modified with high targeting requirements for the exosome surface. Although TDEs can serve as drug carriers, it is imperative to conduct a thorough and systematic evaluation of their potential safety hazards before considering their application 306. Using exosomes to deliver drugs is affected by internal and external factors, including the source, dosage, and administration route of the exosomes. These factors contribute to the reduced accumulation of exosomes in the liver when higher doses are injected 307. Although natural exosomes are more suitable for clinical treatment, the number of exosomes released by cells is relatively small, and the current method of exosome purification is inefficient. As a result, this approach is challenging to popularize, and many studies have attempted to synthesize exosome-specific nanocarriers for clinical treatment. Additionally, the high-volume production of therapeutic exosomes can be achieved using efficient purification methods. Nonetheless, additional assessments are required for their effective utilization in various cells 48.

Treatment options related to tumor immunity also have limitations. Although previous studies have revealed that exosomes have the potential to assist in developing new cancer vaccines and immunomodulators, there remain many problems in their practical application. Therefore, the selection and modification of cell-derived exosomes to reduce safety hazards and improve prognosis must be considered. As exosomes can affect the formation of TME, in the future, it may also be possible to use exosomes to regulate the formation and properties of TME to interfere with the immune escape of tumors, thereby improving the efficacy of adjuvant therapy. Liu *et al.* 308 reviewed the application of EVs in immunotherapy, suggesting that exosomes can be applied to immune reprogramming strategies and checkpoint blockade. Similarly, Lin *et al.* 309 investigated macrophage-derived cellular vesicles in near-infrared II imaging-guided precise cancer photo-immunotherapy, which identified a new direction for studying exosomes in the field of tumor immunotherapy.

Although some exosomes can be directly used as drugs to form tumor treatment regimens, their number and type are few compared to those of other cancer types. Accordingly, this area has multiple shortcomings, rendering it conducive to future advancements. The investigation of exosome-mediated miRNA transport has garnered significant attention. Consequently, future investigations of miRNA distribution, activity, and expression can yield novel insights into tumor diagnosis and therapy. Exosome extraction and analysis may substitute invasive body fluid collection for early diagnosis and prognosis evaluation in various disorders, as the understanding of exosomes improves.

## Figures and Tables

**Table 1:** Biomarkers used in exosomes for breast cancer diagnosis.

Biomarker	Molecular category	Reference
miR-21	miRNA	^146^
miR-1246		
miR-101	miRNA	^147^
miR373		
miR-106a-363	miRNA	^148^
CEA	Protein	^144,145^
CA153		

**Table 2:** Biomarkers used in exosomes for lung cancer diagnosis.

Biomarker	Molecular category	Reference
miR-17-3p	miRNA	^157^
miR-21		
miR-106a		
miR-146		
miR-155		
miR-191		
miR-192		
miR-203		
miR-205		
miR-210		
miR-212		
miR-214		
miR-378a	miRNA	^158^
miR-379		
miR-139-5p		
miR-200b-5p		
miR-320d	miRNA	^159^
miR-320c		
miR-320b		
miR-181-5p	miRNA	^160^
miR-30a-3p		
miR-30e-3p		
miR-361-5p		
miR-10b-5p		
miR-15b-5p		
miR-320b		
miR-146a-5p	miRNA	^153^
miR-486-5p		
miR-17-5p	miRNA	^161^
circSATB2	circRNA	^162^
CEA	Protein	^161^
CYFRA21-1		
SCCA		
LRG1	Protein	^163^
CD91	Protein	^164^
Tim-3	Protein	^165^
Galectin-9		
TP53	DNA	^166^
PKD1		
ALK		
GAS5	DNA	^167^

**Table 3:** Biomarkers used in exosomes for CRC diagnosis.

Biomarker	Molecular category	Reference
miR-17-92a	miRNA	^168^
miR-19a		
let-7a	miRNA	^172^
miR-1229		
miR-1246		
miR-150		
miR-21		
miR-223		
miR-23a		
miR-638	miRNA	^173^
miR-125a-3p	miRNA	^169^
miR-320c		
miR-27a	miRNA	^174^
miR-193a-5p	miRNA	^175^
miR-23b	miRNA	^176^
miR-99b-5p	miRNA	^177^
miR-150-5p		
miR-760	miRNA	^178^
miR-29a		
miR-92a		
miR-17-5p	miRNA	^179^
miR-92a-3p		
miR-548c-5p	miRNA	^180^
miR-638	miRNA	^181^
miR-217	miRNA	^182^
lncRNA	lncRNA	^182^
LNCV6_116109	lncRNA	^183^
LNCV6_98390		
LNCV6_38772		
LNCV_108266		
LNCV6_84003		
LNCV6_98602		
FOXD2-AS1	lncRNA	^184^
NRIR		
XLOC_009459		
NNT-AS1	lncRNA	^185^
circLPAR1	circRNA	^186^
circ_0004771	circRNA	^187^
CPNE3	DNA	^188^
QSOX1	DNA	^189^

**Table 4:** Biomarkers used in exosomes for PCa diagnosis.

Biomarker	Molecular category	Reference
miR-141	miRNA	^193^
miR-375	miRNA	^194^
miR-107	miRNA	^193^
miR-574-3p		
miR-19a	miRNA	^168^
PCA-3	DNA	^54^
TMPRSS2		

**Table 5:** Biomarkers used in exosomes for other cancer diagnoses.

Biomarker	Molecular category	The corresponding cancer	Reference
miR-191	miRNA	Pancreatic cancer	^203^
miR-21			
miR-451a			
GPC1	Protein	Pancreatic cancer	^68^
MIF	Protein	Pancreatic cancer	^204^
miR-21	miRNA	Esophageal cancer	^205^
miR-155	miRNA	Esophageal cancer	^206-208^
miR-652-5p			
miR-339-5p			
Lnc-PCAT1	lncRNA	Esophageal cancer	^209-211^
Lnc-UCA1			
Lnc-POU3F3			
Lnc-ESCCAL-1			
Lnc-NR_039819			
Lnc-NR_036133			
Lnc-NR_003353			
Lnc-PEG10			
Lnc-ENST00000416100.1			
Lnc-ENST00000442416.1			
miR-17	miRNA	Melanoma	^212^
miR-19a			
miR-21			
miR-126			
miR-149			
PD-L1	Protein	Melanoma	^213^
Caveolin-1	Protein	Melanoma	^214^
miR-25-3p	miRNA	Osteosarcoma	^215,216^
miR-21	miRNA	Thyroid cancer	^217^
miR-181a-5p			
Dynamin 3 (DNM3) mRNA	miRNA	Glioblastoma multiforme	^218^
P65mRNA			
miR-21	miRNA	Laryngeal cancer	^219^
Hox transcript antisense intergenic RNA (HOTAIR)	lncRNA	Laryngeal cancer	^219^
lncRNA	lncRNA	Cervical cancer	^220^
Claudin	Protein	Ovarian cancer	^221^
